# Effects of non-exciton components excited by broadband pulses on quantum beats in a GaAs/AlAs multiple quantum well

**DOI:** 10.1038/srep41496

**Published:** 2017-01-27

**Authors:** Osamu Kojima, Yuki Iwasaki, Takashi Kita, Kouichi Akahane

**Affiliations:** 1 Department of Electrical and Electronic Engineering, Graduate School of Engineering, Kobe University, 1-1 Rokkodai, Nada, Kobe 657-8501, Japan; 2 National Institute of Information and Communications Technology, 4-2-1 Nukui-kitamachi, Koganei, Tokyo 184-8795, Japan

## Abstract

In this study, we report the effect of the excitation of non-exciton components caused by broadband pulses on quantum beat oscillation. Using a spectrally controlled pump pulse, a long-lived oscillation is clearly observed, and the pump-power dependence shows the suppression of the dephasing rate of the oscillation. Our results from incoherent carrier generation using a continuous wave laser demonstrate that the non-exciton components behaving as free carriers increase the oscillation dephasing rate.

Coherent exciton oscillations, such as quantum beats and Bloch oscillations, have been extensively studied to understand the ultrafast exciton dynamics^1^,^2^,^3^,^4^,^5^,^6^,^7^,^8^,^9^, the interaction of excitons with phonons^10^,^11^,^12^,^13^,^14^,^15^,^16^,^17^,^18^, the determination of the splitting energies in inhomogeneous systems^19^,^20^,^21^, and applications of ultrafast devices^22^,^23^,^24^. However, one problem has been the difficulty of generating a clear and long-lived oscillation. In general, to generate a strong oscillation, the pump pulse needs to be tuned to the central energy of the two exciton states^6^,^12^. For example, when an ultrashort pulse laser with a broad spectral width is tuned to the central energy of heavy-hole (HH) and light-hole (LH) excitons split by a quantum size effect, an HH-LH exciton quantum beat with the maximum amplitude is obtained. In this case, however, because the spectrum of the pump laser has various components, including the excitons, the non-exciton components are also excited. For instance, several four-wave-mixing spectra under exciton excitation conditions have exhibited the broad tail around the exciton lines^25^,^26^. These components may act as free carriers and lead to exciton quantum beat dephasing^27^,^28^.

In contrast, pulse spectrum control using a so-called phase mask has also been extensively studied in the field of ultrafast spectroscopy^29^,^30^,^31^,^32^,^33^, e.g., a long-lived oscillation has been observed in a terahertz signal^30^ and the quantum beat in the photon echo spectrum was observed^33^. Further, the coherent control of oscillation dynamics has been reported^34^,^35^. Because the phase mask can excite only the excitons, the effect of non-exciton components on the dephasing of the quantum beats will be filtered, which helps generate a clearer and longer-lived oscillation when using the pump-probe technique. Considering the realization of devices based on the quantum beats including frequency tunable terahertz-wave emitters at room temperature, the long-lived oscillation at low temperature should be obtained and the generation condition should be clarified. Therefore, in this study, we report the exciton quantum beat generated by the spectrally controlled pulse using a double slit. The oscillation of the quantum beat was clearly observed by pumping with spectrally controlled pulses. Furthermore, the oscillation-dephasing rate pumped by the spectrally controlled pulse was smaller than that pumped by an uncontrolled broadband pulse, which demonstrates that non-exciton components generated by the broadband pulse increase the dephasing rate of the quantum beat. The origin of the quantum beat dephasing due to the non-exciton components generated by the broadband pulse excitation are discussed using the results of the pump-power and pump-energy dependence and combination with continuous wave (CW) laser excitation.

Figure 1(a) shows the typical result of spectrum control of the laser pulse, where the original shape (uncontrolled) is indicated by the dashed curve. The energy positions of the two peaks nearly agree with the HH and LH exciton energies in the sample. The temporal pulse shapes of the spectrally controlled and uncontrolled pulses are indicated in Fig. 1(b) by the solid and dashed curves, respectively. The dotted curve demonstrates the Fourier transformation (FT) profile of the controlled laser spectrum in Fig. 1(a). While the pulse shape of the uncontrolled spectrum has a single peak described approximately by a sech^2^ function, the spectrally controlled pulse has several peaks. The temporal positions of these peaks nearly agree with the Fourier profile. However, the asymmetric profile may be attributed to the distorted spectral shape.

The pump-probe signals observed by the controlled and uncontrolled pulses are depicted in Fig. 1(c) by the solid and dashed curves, respectively. The excitation power was the same in both measurements. The oscillatory structure caused by the quantum beat appears in both signals. However, while the oscillation period is nearly same in the two signals, the oscillation pumped by the controlled pulse is clearer and lasts longer. The period is approximately 260 fs, which corresponds to the heavy-hole and light-hole exciton splitting energy of 16 meV in this sample. Since this energy does not agree with the energies of phonons, coherent phonons generated by an impulsive stimulated Raman scattering mode hardly contribute to the signals. To clarify the oscillation characteristics, the oscillation component was extracted from the signal after 0 ps by eliminating the background and was analyzed using a harmonic damped oscillation model. Even though a model with three components based on the optical Bloch equation should be used for this analysis^36^,^37^, the observed signal shape was too complicated to be analyzed in that way. Therefore, the evaluated values can be qualitatively compared, but the absolute value cannot be discussed. The evaluated amplitudes and dephasing rates are listed in Table 1. The amplitude from the controlled pump is larger than that by the uncontrolled pump, and the damping factor is smaller for the former. This result clearly demonstrates the advantage of using a spectrally controlled pulse to generate the quantum beat oscillation. In particular, considering that the excitation density for each exciton by the controlled pump is higher than that by the uncontrolled pulse, the difference in the damping factor illustrates that the excitation of the non-exciton components induces the dephasing of the oscillation.

The pump-power dependence was measured to demonstrate the effect of the density of coherent excitons on quantum beat dephasing. In Fig. 2(a), the oscillatory components extracted from the signal with the background are depicted by open circles. The solid curves are the fitting results based on the analysis mentioned above. The analyzed amplitude and damping rate are plotted in Fig. 2(b) by the open and closed circles, respectively. The dashed line indicates the dephasing rate by the uncontrolled pump pulse at 1 μJ/cm^2^. While the amplitude increases proportionally to the pump power, the dephasing rate is nearly constant in this pump power region, which is a key point in this study. When the spectrally controlled pump pulse is used, the number of generated excitons is larger than that generated by the uncontrolled broadband pulse under the same excitation power condition. Nevertheless, the dephasing rate is suppressed by the controlled pump pulse. These results indicate that the non-exciton components pumped by the uncontrolled broadband pulse lead to the dephasing of the quantum beats and that exciton-exciton scattering is not major factor to increase the dephasing rate of quantum beat.

Next, to show the resonant excitation effect, the pump energy dependence was measured, as shown in Fig. 3(a). The laser profiles are indicated in Fig. 3(b). The order of the profiles from the top to bottom is same in Fig. 3(a). Here, the energy separation of the two laser peaks was kept at approximately 15 meV. The amplitude was altered with the pump energy. The evaluated amplitude is plotted as a function of the pump energy in Fig. 3(b). Although the HH exciton is almost off-resonant condition at 1.576 eV, the strong oscillation was observed. On the other hand, the excitation at 1.578 eV is also off-resonant excitation of LH exciton. Therefore, the excitation of the LH exciton may be more important to induce the quantum beat. This result demonstrates that the observed oscillatory structure arises from the exciton quantum beat. That is, the virtual exciton^38^ does not relate to the oscillation. Furthermore, in this measurement, the phase change noted in Ref.^33^. was no observed. We considered that this difference originates from the measurement technique; the pump-probe and photon echo.

Here, the possible factors increasing the dephasing rate by broadband pulse excitation are discussed. At first, the effects by HH-LH mixing and the intersubband transition from the LH to HH states are denied, because these factors are based on the structure; unless the effects by free carries, interaction with the continuum carriers, and so on are considered, these factors hardly change.

Then, finally, to show that the non-exciton components pumped by the broadband pulse act as the background carrier for the quantum beat oscillation, we measured the oscillation under CW laser excitation. The CW laser generates incoherent carriers at 1.85 eV. As shown in Fig. 4(a), as the excitation power increases, the signal profile changes. Here, the signals are not rescaled, and there is no offset. The result for the CW excitation looks similar to that of the broadband pulse in Fig. 1(c); the signal intensity in the positive time region is higher than that in the negative time region.

An analysis of the oscillatory component was performed as shown in Fig. 4(b). The solid curves indicate the fitting results. The evaluated dephasing rates are listed in Table 2. The rate increases by CW excitation and the value is saturated at 3 mW. The carriers generated by the CW laser are incoherent; therefore the interaction with excitons generated by the resonant pulse causes exciton dephasing. That is, the non-exciton components pumped by the broadband pulse also induce exciton dephasing, which increases the dephasing rate of the quantum beat. There is a possibility that the phonon scattering induced by the broadband pulse excitation increases the quantum beat dephasing. It is difficult to perfectly deny this factor. However, considering the temperature dependence of the dephasing rate^18^, which does not agree with that of the thermal phonons, the contribution of the phonon is very small. Moreover, although the number of the carriers generated by the CW 1 mW excitation is small, the large change of the dephasing rate was observed. We consider that this fact supports elimination of the factor of phonon scattering.

In summary, we have investigated the effects of the non-exciton components included in the spectrum of a broadband pump pulse. When a spectral-shape controlled pulse was used to resonantly excite the HH and LH excitons, the quantum beat oscillation was clearly observed and the dephasing rate was suppressed. Moreover, the incoherent carriers generated by the CW laser lead to quantum beat dephasing and show the same behavior as the non-exciton components generated by a broadband pulse. This result indicates that the carriers generated by non-exciton component of the laser pulse changes to the free carriers inducing the dephasing of quantum beat. In this work, the laser widths in our measurements were much broader than the line width of HH exciton. Therefore, we can expect that the further long dephasing time of the quantum beat is realized by narrowing the laser spectrum.

## Methods

### Sample structure

The sample used in this study was a (GaAs)_35_/(AlAs)_35_ multiple quantum well (MQW) grown on a (001) GaAs substrate by molecular-beam epitaxy, where the subscript denotes the constitution layer thickness of a monolayer unit of 0.283 nm. The MQW period was 50. The homogeneous and inhomogeneous broadening factors estimated from the photoluminescence spectrum at 4 K were 0.6 and 0.8 meV, respectively. The estimation method was described in Ref.^18^.

### Quantum beat measurement

**Figure 5:**
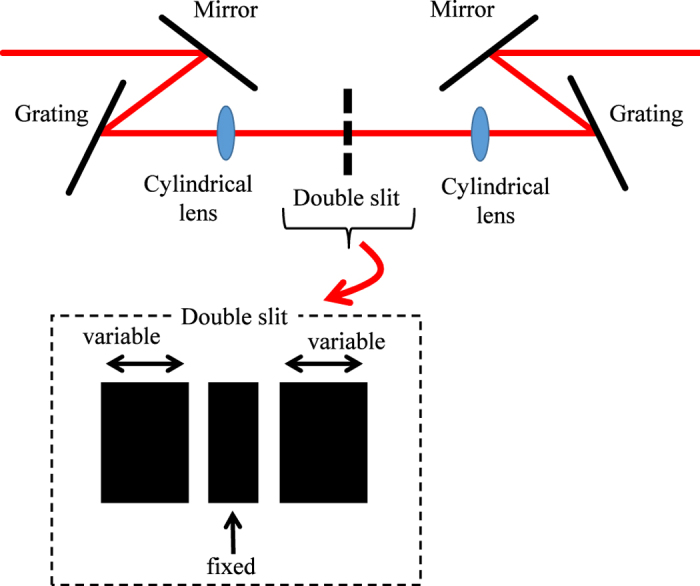
System to control the spectrum of the pump pulse consisting of two gratings, two cylindrical lenses, and a double slit.

The quantum beat was measured using a time-resolved reflection-type pump-probe technique. The measurement temperature was 4 K. The laser source used was a mode-locked Ti:sapphire pulse laser, delivering an approximately 90 fs pulse with a repetition rate of 80 MHz. While the probe power was kept at 0.06 μJ/cm^2^, the pump power was changed from 0.1 to 2 μJ/cm^2^. The reflection change is induced by the change of the internal electric field and Pauli blocking^39^. A schematic of the system controlling the spectrum of the pump pulse is shown in Fig. 5. The system was constructed using two gratings with 1800 lines/mm, two cylindrical lenses with a focal length of 150 mm, and a double slit. In the double slit, the width of the center part was fixed, and the slit widths were changed by changing the position of both side blades. The pump beam was orthogonally polarized to the probe beam in order to eliminate its contribution. The pump beam was chopped at 2 kHz, and the intensity of the reflected probe beam was modulated. The probe intensity detected by the Si photodiode was amplified using a lock-in amplifier.

## Additional Information

**How to cite this article**: Kojima, O. *et al*. Effects of non-exciton components excited by broadband pulses on quantum beats in a GaAs/AlAs multiple quantum well. *Sci. Rep.* **7**, 41496; doi: 10.1038/srep41496 (2017).

**Publisher's note:** Springer Nature remains neutral with regard to jurisdictional claims in published maps and institutional affiliations.

## Figures and Tables

**Figure 1:**
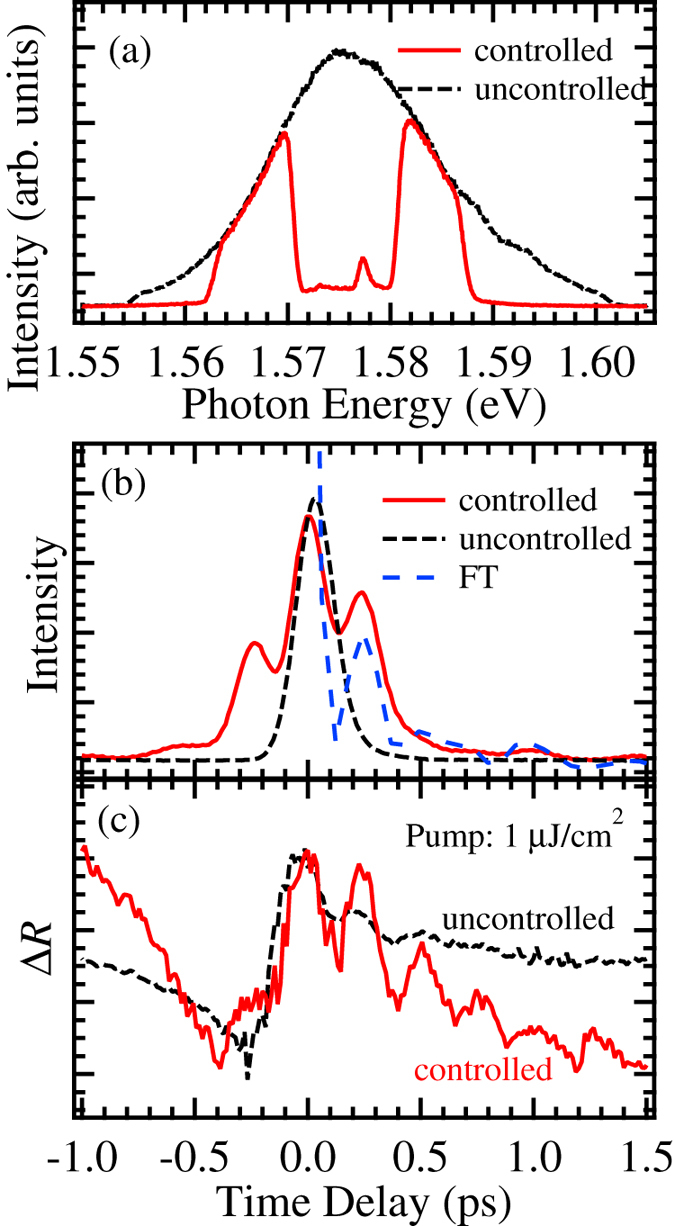
(**a**) Laser spectra of controlled (solid curve) and uncontrolled (dashed curve) pump pulses. (**b**) Temporal profiles of the spectrally controlled (solid curve) and uncontrolled (dashed curve) pump pulses, and the FT profile of the controlled pulse in (**a**). (**c**) Pump-probe signals measured by the controlled (solid curve) and uncontrolled (dashed curve) pump pulses.

**Figure 2:**
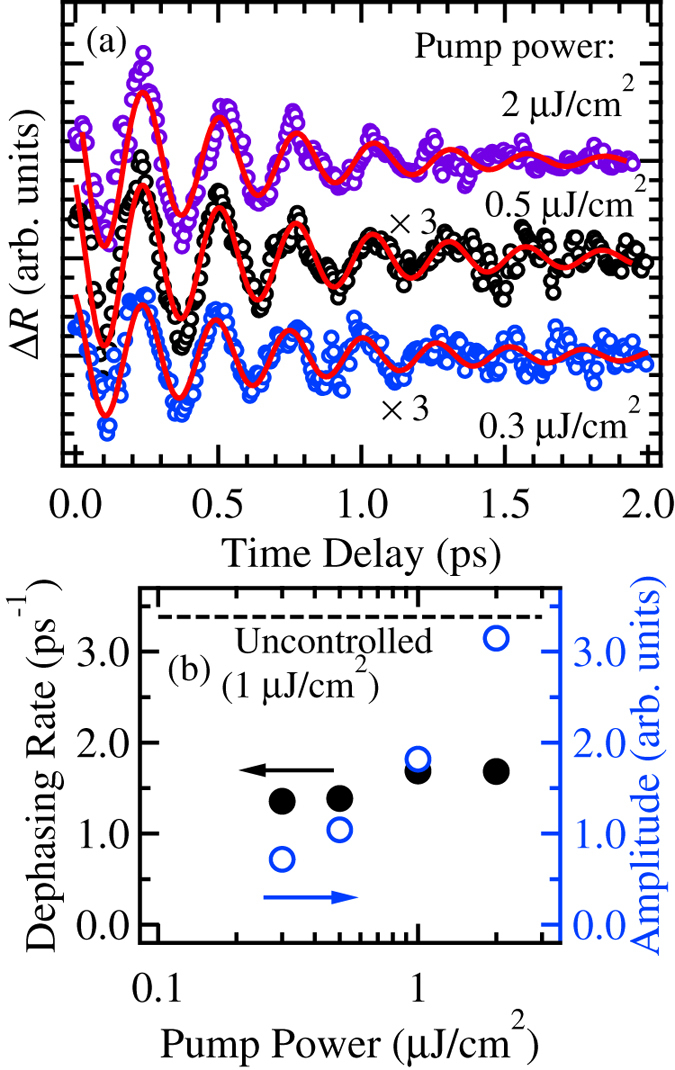
(**a**) Dependence of the oscillatory component using a spectrally controlled pump pulse on the pump power depicted by the open circles. The solid curves indicate the fitting results. (**b**) The evaluated amplitude and dephasing rate are plotted as a function of the pump power by the closed and open circles, respectively. The dashed line indicates the dephasing rate of an uncontrolled pump pulse at 1 μJ/cm^2^.

**Figure 3:**
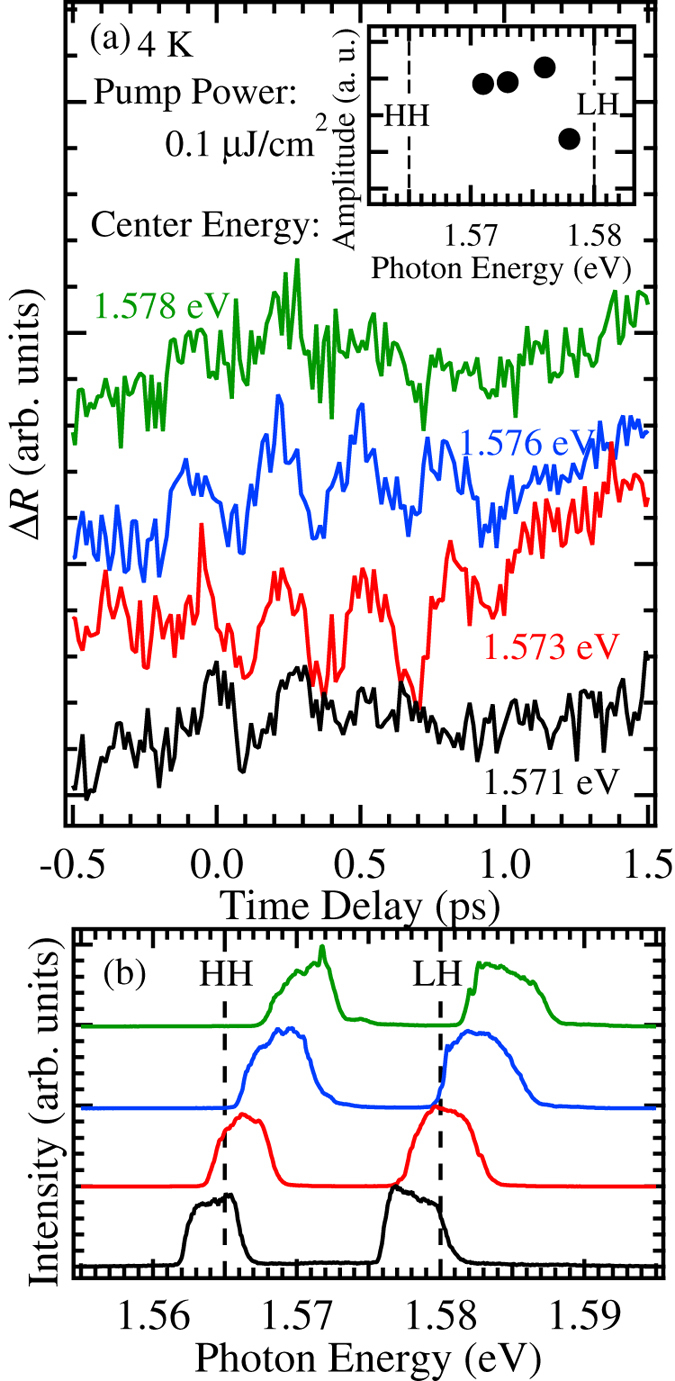
(**a**) Dependence of the oscillatory component on the pump energy for a pump power of 0.1 μJ/cm^2^. The analyzed amplitude was plotted as a function of pump energy in the inset. (**b**) The laser profiles used in (**a**). The dashed lines indicate the exciton energies.

**Figure 4:**
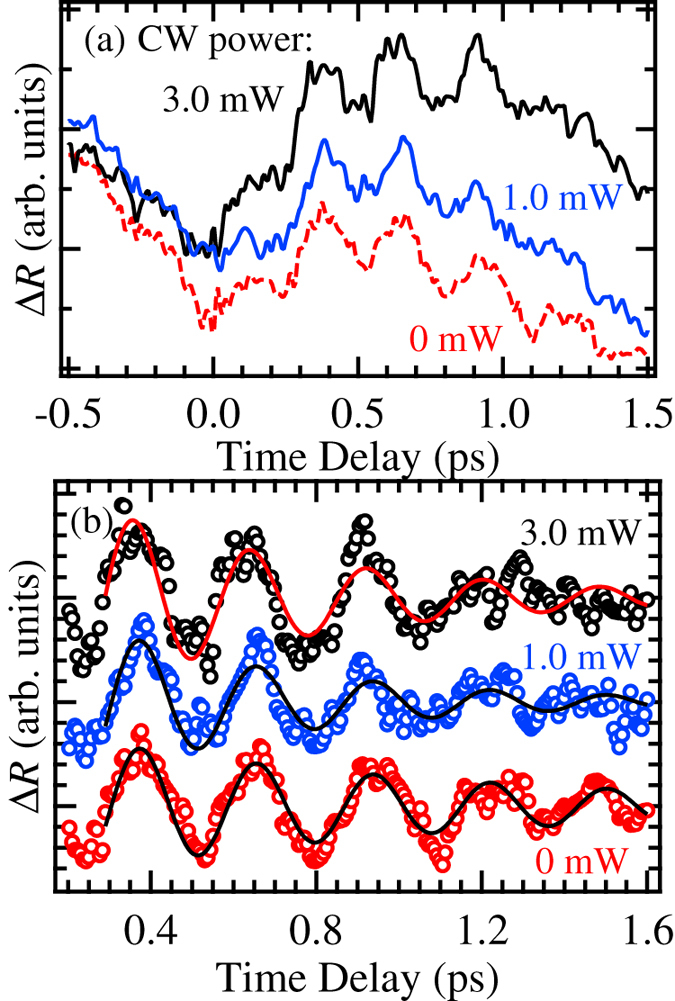
(**a**) Dependence of the pump-probe signal on the CW excitation power. (**b**) The oscillatory structures are plotted with open circles. The solid curves indicate the fitting results.

**Table 1:** Evaluated amplitudes and dephasing rates.

	Amplitude	dephasing rate (ps^−1^)
controlled	1.818	1.690
uncontrolled	0.559	3.383

**Table 2:** Evaluated dephasing rates.

CW power (mW)	Dephasing rate (ps^−1^)
0	1.063 ± 0.113
1	1.925 ± 0.222
3	1.708 ± 0.192
